# Bedside interpretation of cerebral energy metabolism utilizing microdialysis in neurosurgical and general intensive care

**DOI:** 10.3389/fneur.2022.968288

**Published:** 2022-08-10

**Authors:** Carl-Henrik Nordström, Axel Forsse, Rasmus Peter Jakobsen, Simon Mölström, Troels Halfeldt Nielsen, Palle Toft, Urban Ungerstedt

**Affiliations:** ^1^Department of Neurosurgery, Odense University Hospital, Odense, Denmark; ^2^Department of Neurosurgery, Rigshospitalet, Copenhagen, Denmark; ^3^Department of Anesthesiology and Intensive Care, Odense University Hospital, Odense, Denmark; ^4^Department of Physiology and Pharmacology, Karolinska Institute, Stockholm, Sweden

**Keywords:** microdialysis, cerebral energy metabolism, ischemia, mitochondrial dysfunction, lactate, pyruvate, cardiac arrest, resuscitation

## Abstract

The microdialysis technique was initially developed for monitoring neurotransmitters in animals. In 1995 the technique was adopted to clinical use and bedside enzymatic analysis of glucose, pyruvate, lactate, glutamate and glycerol. Under clinical conditions microdialysis has also been used for studying cytokines, protein biomarkers, multiplex proteomic and metabolomic analyses as well as for pharmacokinetic studies and evaluation of blood-brain barrier function. This review focuses on the variables directly related to cerebral energy metabolism and the possibilities and limitations of microdialysis during routine neurosurgical and general intensive care. Our knowledge of cerebral energy metabolism is to a large extent based on animal experiments performed more than 40 years ago. However, the different biochemical information obtained from various techniques should be recognized. The basic animal studies analyzed brain tissue homogenates while the microdialysis technique reflects the variables in a narrow zone of interstitial fluid surrounding the probe. Besides the difference of the volume investigated, the levels of the biochemical variables differ in different compartments. During bedside microdialysis cerebral energy metabolism is primarily reflected in measured levels of glucose, lactate and pyruvate and the lactate to pyruvate (LP) ratio. The LP ratio reflects cytoplasmatic redox-state which increases instantaneously during insufficient aerobic energy metabolism. Cerebral ischemia is characterized by a marked increase in intracerebral LP ratio at simultaneous decreases in intracerebral levels of pyruvate and glucose. Mitochondrial dysfunction is characterized by a moderate increase in LP ratio at a very marked increase in cerebral lactate and normal or elevated levels of pyruvate and glucose. The patterns are of importance in particular for interpretations in transient cerebral ischemia. A new technique for evaluating global cerebral energy metabolism by microdialysis of the draining cerebral venous blood is discussed. In experimental studies it has been shown that pronounced global cerebral ischemia is reflected in venous cerebral blood. Jugular bulb microdialysis has been investigated in patients suffering from subarachnoid hemorrhage, during cardiopulmonary bypass and resuscitation after out of hospital cardiac arrest. Preliminary results indicate that the new technique may give valuable information of cerebral energy metabolism in clinical conditions when insertion of an intracerebral catheter is contraindicated.

## Introduction

Our knowledge regarding cerebral energy metabolism during physiological and pathological conditions is to a large extent based on animal experiments performed more than 40 years ago. Many of these studies were accomplished in a laboratory lead by professor Bo K Siesjö at Lund University Hospital, Sweden, and many important principles were summarized in a textbook and in neurosurgical journals (1–4). Since then, introduction of new analytical techniques has increased our knowledge considerably (5, 6) but the basic biochemical patterns established in the original studies are still relevant for intensive care. For many years it has been impossible to monitor cerebral energy metabolism bedside during clinical conditions. However, the development of microdialysis opened up this possibility.

The microdialysis technique was initially developed for monitoring neurotransmitters in the animal brain (7, 8). In the late 1980s monitoring of the human brain was explored in occasional patients (9–11). In 1995, CMA Microdialysis (Stockholm, Sweden; present manufacturer M Dialysis, Stockholm, Sweden) introduced a sterile microdialysis catheter, a simple microdialysis pump and an analyzer for bedside enzymatic measurements of variables related to cerebral energy metabolism (glucose, pyruvate, lactate) as well as glutamate and glycerol.

Microdialysis is an open technique that allows analysis of an innumerable number of variables. Under clinical conditions it has been used for studying cytokines and protein biomarkers, performing multiplex proteomic and metabolomic analyses (12–15) as well as for pharmacokinetic studies and evaluation of blood-brain barrier function (16–20). This review will focus on the possibilities and limitations of the microdialysis technique when used in critical care for analysis and interpretation cerebral energy metabolism (21).

## The microdialysis technique

The basic principles of microdialysis are well-known. In 1991 Urban Ungerstedt summarized the principles and applications when the technique is used for studies in animals and man (9). We will discuss some aspects of particular importance when cerebral microdialysis is used as a routine technique in neurosurgical as well as in general intensive care utilizing the recently developed technique of performing microdialysis of the draining cerebral blood in the internal jugular vein.

In clinical microdialysis the technique is standardized to permit comparison of data from different centers. During routine microdialysis in neurocritical care a microdialysis catheter with a membrane length of 10 mm and a cut off level of 20 kDa is utilized and perfused at a rate of 0.3 μl/min. The perfusates are collected in microvials and bedside biochemical analysis is usually performed every 60 min. If more frequent sampling is desired, perfusion rate is often increased to 2.0 μl/min.

Due to incomplete recovery, the data obtained for the variables (e.g., glucose, pyruvate, lactate, glutamate, glycerol) does not show their true interstitial levels. During intracerebral microdialysis the relative recovery for these variables has been shown to be approximately 70% at a perfusion rate of 0.3 μl/min and 20–30% at 2.0 μl/min (22). For scientific purposes and analyses of large molecules microdialysis membranes with a higher cut off (100 kDa) have been utilized. It has been shown that the dialysate obtained with these catheters may be used for measurements of routine biochemical variables as well (23, 24).

The time-delay inherent in the microdialysis technique should be noted: the microvial contains the perfusion fluid collected since the previous analysis—usually performed 60 min earlier. However, due to the design of the microvial used in clinical microdialysis the perfusate analyzed usually represents the biochemical composition of the interstitial fluid collected during the last 10 min period. If frequent samples are analyzed another time-delay should be recognized: the delay for the perfusion fluid to pass from the dialysis membrane to the collecting microvial. This problem should be considered also when perfusion rate is relatively high (e g., 2.0 μl/min) (25).

**Figure 1:**
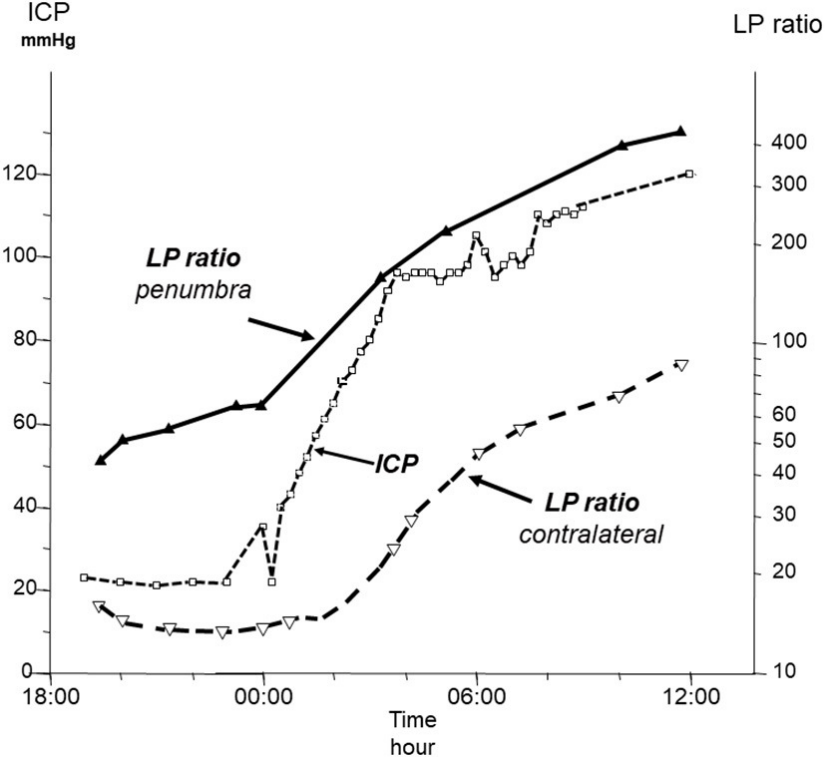
Time course of intracranial pressure (ICP) and lactate/pyruvate (LP) ratio in the penumbra zone of a focal brain lesion and in the contralateral hemisphere in a patient with a fatal traumatic injury. Data from (27).

The microdialysis catheter collects interstitial fluid from a very narrow zone surrounding the probe and the biochemical patterns obtained are very different if the microdialysis catheter is positioned in the penumbra zone surrounding a focal lesion, in the hemisphere distant from the lesion or in the contralateral hemisphere (26). During neurocritical conditions, the perturbation of cerebral energy metabolism is initially often local. Accordingly, an early warning of impending biochemical deterioration is obtained provided the catheter is positioned correctly (Figure 1) (27–30). Thus, based on bedside biochemical information active interventions may be initiated to limit or prevent tissue damage (31). Inversely, an irrelevant alarming biochemical pattern may be obtained if a very local, clinically non-significant lesion or hematoma has developed at the tip of the microdialysis probe. This problems is generally solved by relating the biochemical pattern to the position of the catheter defined from repeat CT-scanning.

In many severe clinical conditions, it would be valuable to compare the very local biochemical data obtained from intracerebral microdialysis to information reflecting “global” cerebral energy metabolic state. Hence, it is of interest to explore whether microdialysate obtained from a catheter positioned in the draining cerebral venous blood might be used for this purpose. In particular, such a technique could be of importance in patients with a global perturbation of cerebral energy metabolism in conditions when insertion of an intracerebral catheter would be difficult or impossible. In these patients it might be possible to position the microdialysis catheter in the bulb of the internal jugular vein. Such clinical studies were preceded by animal experiments to evaluate whether microdialysis of the draining venous blood reflected intracerebral energy metabolism in a meaningful way. However, cerebral venous drainage is very different in different species. In primates, like in man, cerebral blood is mainly drained *via* the internal jugular vein but in swine the main cerebral venous outflow is through the paraspinal venous network (32, 33). Accordingly, in the experimental studies in pigs presented below the venous microdialysis catheters were inserted into the superior sagittal sinus.

## Variables reflecting cerebral energy metabolism

As discussed previously, cerebral energy metabolism has been explored in detail during various patho-physiological conditions in experimental studies (1–4). Data obtained utilizing microdialysis should be interpreted in the light of these experiences. However, it should be noticed that the biochemical information obtained from various techniques may be very different. The animal studies referred to were all performed by analyzing biochemical variables in brain tissue homogenates while microdialysis reflects the pattern in a narrow zone of interstitial fluid surrounding the probe. Besides the difference in volume investigated, the levels of the biochemical variables differ markedly in various tissue compartments—e.g., glutamate concentration is under normal conditions very low in cerebral interstitial fluid (<1 μmol/L), relatively high in astrocytes (2 mmol/L), high in the postsynaptic (10 mmol/L), very high in the presynaptic compartment (100 mmol/L) and approximately 30–80 μmol/L in cerebral capillary blood (34).

Lactate and pyruvate are water soluble. Due to monocarboxylate transporters (MCTs) they equilibrate rapidly across cellular membranes. MCTs are proton linked membrane carriers present in all tissues and involved in the transport of various monocarboxylates such as lactate, pyruvate and ketone bodies (35–39). Out of the total family of 14 members three isoforms (MCT1, MCT2, MCT4) have been described in the brain (40). The driving forces for the transport of the monocarboxylates are obtained from the concentration differences over the cellular membranes. The transport is consequently characterized as facilitated diffusion (41). Due to the MCTs, it is adequate to regard the levels of lactate and pyruvate in cerebral interstitial fluid as a measure of their cytoplasmatic levels.

**Figure 2:**
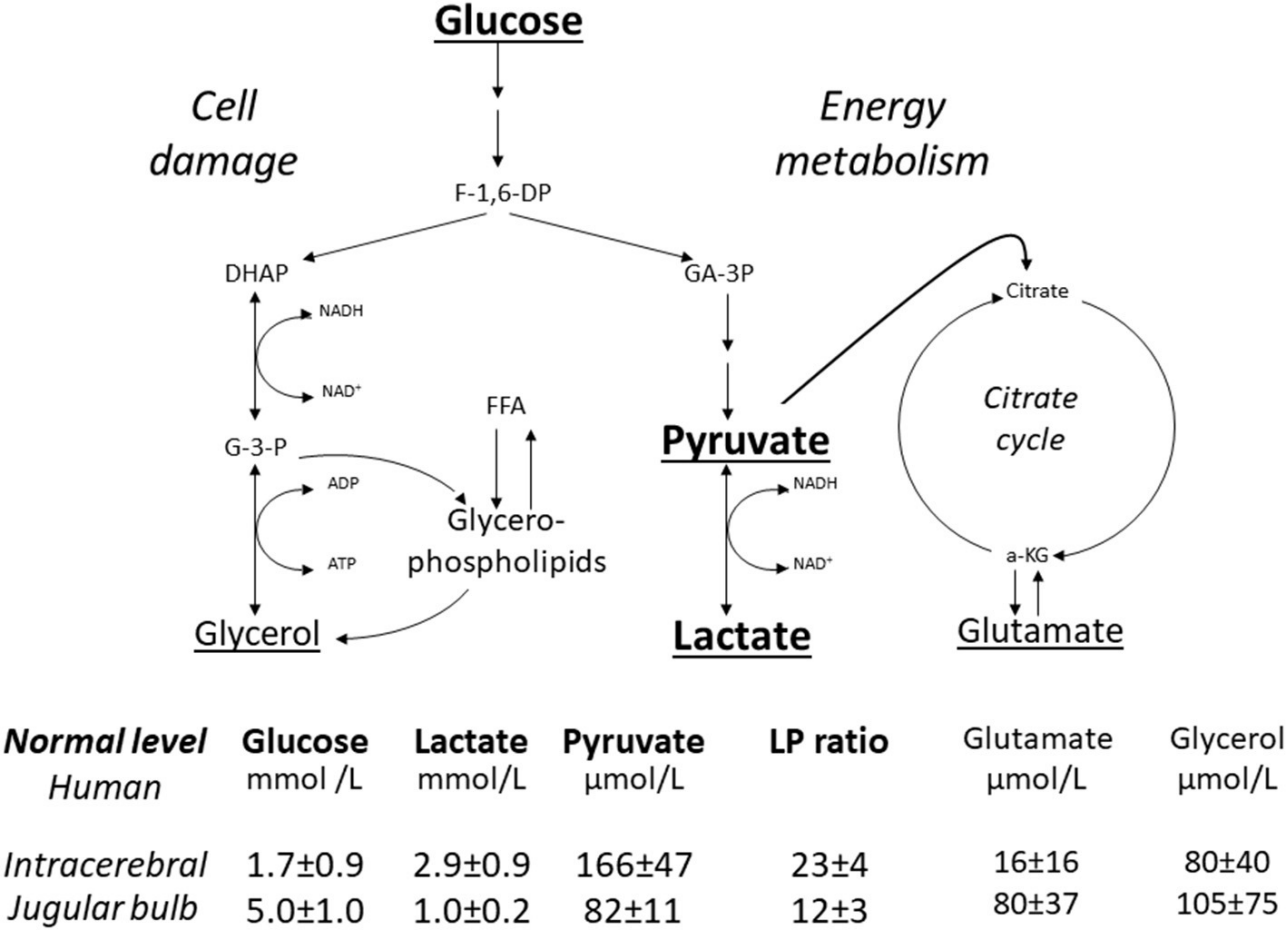
Schematic diagram of cerebral intermediary metabolism, with a focus on the glycolytic chain (glucose, pyruvate, lactate) and its relation to glycerol and glycerophospholipids and to the citric acid cycle (Krebs cycle). F-1,6-DP, fructose-1,6-diposphate; DHAP, dihydroxyacetone-phosphate; GA-3P, glyceraldehyde-3-phosphate; G-3-P, glycerol-3-phosphate; FFA, free fatty acids; α-KG, α-ketoglutarate. Underlined metabolites are measured bedside with enzymatic techniques. References levels of the various metabolites for normal human brain obtained from (42). Reference levels for jugular venous blood obtained from (43). Reference values are given as Mean ± SD.

Figure 2 gives a simplified illustration of the biochemical variables measured during routine intracerebral microdialysis as well as their reference values in normal human brain and cerebral venous blood measured at the level of the jugular bulb (42, 43). In this review we focus on the variables directly related to cerebral energy metabolism, i.e., glucose, pyruvate and lactate.

Brain tissue may use various substrates for energy metabolism but under normal conditions glucose constitutes the sole substrate of importance (1). In the cytosol, glucose is degraded to pyruvate (glycolysis) with a net yield of 2 ATP for each molecule of glucose. The major part of pyruvate enters the citric acid cycle in the mitochondria and is completely degraded to CO_2_ and H_2_O with a net yield of another 36 ATP. Depending on the prevailing cytoplasmatic redox conditions, part of the pyruvate is converted to lactate.

The lactate/pyruvate (LP) ratio reflects cytoplasmatic redox-state, which can be expressed in terms of the lactate dehydrogenase equilibrium:


(1) $$\begin{array}{r} \frac{\left[\text{NADH}\right]\left[{\text{H}}^{+}\right]}{\left[\text{NAD}\right]}=\frac{\left[\text{Lactate}\right]}{\left[\text{Pyruvate}\right]}\times K_{\text{LDH}} \end{array}$$

The LP ratio gives information regarding the efficacy of cerebral oxidative energy metabolism. The ratio increases during deficient oxygen delivery (ischemia, hypoxia) and mitochondrial dysfunction (44–48). However, insufficient supply of glucose does not influence the cytoplasmatic redox-state (49, 50). Even during profound hypoglycemia resulting in devastating cell damage LP ratio is only slightly increased (51).

The physiological and biochemical changes during a gradual decrease in cerebral blood flow (CBF) are of particular importance during critical care. At a normal hemoglobin concentration of 150 g/L and 95% saturation, arterial blood contains about 9 μmol of oxygen per mL and arterio-venous oxygen difference is about 3 μmol/mL. Thus, if CBF is reduced to about 1/3 theoretically all oxygen will be extracted. For glucose the following approximate levels have been described: arterial concentration 5.1 μmol/mL; venous concentration 4.6 μmol/mL; arterio-venous glucose difference 0.5 μmol/mL (1). Accordingly, during a gradual decrease in CBF oxygen supply to the brain will be insufficient before the supply of substrate is seriously jeopardized (1).

Consequently, during a reduction of CBF glucose will not be the limiting factor for maintenance of aerobic energy metabolism and insufficient glucose supply will not cause increase of the LP ratio. It has recently been suggested that the injured brain would benefit from exogenous lactate supplementation (52, 53). However, it has been shown that the hypothesis is not supported by basic physiological and biochemical facts (5, 6, 39). In addition, lactate flooding has the potential to inhibit glycolytic, glycogenolytic, and pentose shunt fluxes, further compromising the brain's ability to upregulate cytosolic ATP production, control ionic gradients and extracellular neurotransmitter glutamate levels, and manage oxidative stress, particularly during hyperglycolysis or mitochondrial dysfunction (5).

## Biochemical patterns during increased energy metabolism

Hypermetabolism and hyperglycolysis are defined as a significant increase in cerebral metabolic rate above normal causing an increase in cerebral glycolytic rate. Cerebral hypermetabolism may occur during physiological as well as pathological conditions. The steady state levels of the chemical variables related to energy metabolism do not directly reveal metabolic rate but it is important to define the biochemical patterns expected in various hypermetabolic conditions.

### Hypermetabolism studied in brain tissue homogenates

Cerebral hypermetabolism has been extensively studied in the rat. During immobilization stress CBF and cerebral metabolic rate for oxygen (CMRO_2_) increased by about 40 % after 5 min (54). Analyses of glycolytic intermediates indicated a marked increase in glycolytic rate but the changes in the levels of lactate and pyruvate (and LP ratio) did not change significantly (55). In another rat model intraperitoneal (*i.p*.) administration of amphetamine sulfate (5 mg/kg) caused a marked increase in CBF (4- to 5-fold) and CMRO_2_ (30–40%). After 60 min significant increases in lactate, pyruvate and LP ratio were measured while intracerebral glucose level remained essentially unchanged (56).

In rat experiments the changes in cerebral energy metabolism during sustained epileptic seizures have been studied after administration of *i.v*. bicucullin and *i.p*. homocysteine. Seizures induced by bicuculline were accompanied by a 2- to 3-fold increase in cerebral metabolic rate, very marked increases in intracerebral levels of lactate and LP ratio, a moderate increase in pyruvate and a moderate decrease in glucose (57, 58). Administration of a high dose of homocysteine (11 mmol/kg) caused sustained epileptic seizures and an intracerebral pattern of lactate, pyruvate, LP ratio and glucose similar to that described after *i.v*. bicuculline. A lower dose of homocysteine (7.5 mmol/kg) caused hypermetabolism and hyperglycolysis but no seizures. In this situation modest increases in lactate and pyruvate were obtained leading to a significant but moderate increase in LP ratio while intracerebral glucose level increased (59).

These experimental models show that during moderate cerebral hypermetabolism (immobilization stress) LP ratio is essentially unaffected and cerebral levels of lactate and pyruvate do not change. More pronounced hypermetabolism (homocysteine induced, no seizures) is associated with a moderate increase in LP ratio and modest increases in lactate and pyruvate. During very pronounced hypermetabolism (induced by amphetamine, bicuculline or homocysteine with seizure activity) LP ratio and lactate increases to very high levels while a moderate increase in pyruvate is observed. A moderate decrease in intracerebral glucose occurs after prolonged bicuculline induced seizures. These patterns obtained during experimental studies may be used to interpret clinical situations of suspected hypermetabolism/hyperglycolysis.

### Hypermetabolism/hyperglycolysis and clinical intracerebral microdialysis

The biochemical pattern of hyperglycolysis in normal human cerebral cortex has been described during termination of general anesthesia and extubation (42). These patients were operated for large, benign tumors in the posterior fossa and data from this group have also been used for establishing normal reference levels as shown in Figure 2 (42). After anesthesia and extubation, a pronounced lasting increase in lactate was observed. As pyruvate levels also increased the increase in the lactate/pyruvate ratio was insignificant. A similar pattern of paralleled increases in lactate and pyruvate has been described in patients with a favorable outcome after subarachnoid hemorrhage and interpreted as a sign of beneficial increase in metabolic rate (hyperglycolysis) (60).

There is limited information regarding cerebral energy metabolism during epileptic seizures in patients evaluated by microdialysis. In a study in patients with traumatic brain lesions Vespa et al. (61) reported LP ratio above 40 during seizures and periodic epileptiform discharges but not during electrically non–epileptic periods.

**Figure 3:**
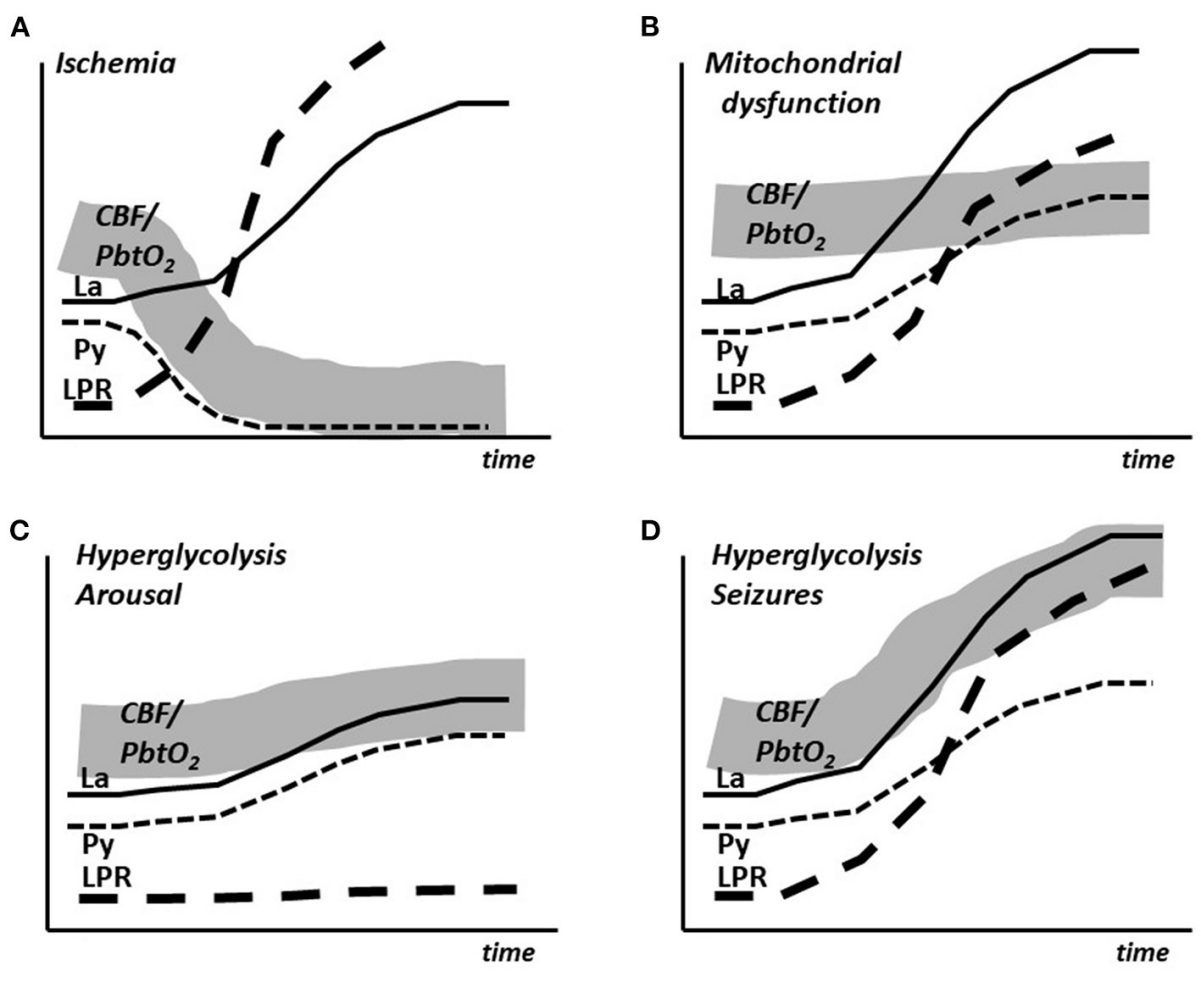
Schematic illustration of the simultaneous changes in cerebral blood flow (CBF), brain tissue oxygen tension (PbtO_2_) and the intracerebral levels of lactate (La), pyruvate (Py) and lactate/pyruvate ratio (LPR) in cerebral ischemia **(A)**, mitochondrial dysfunction **(B)**, arousal **(C)** and epileptic seizures **(D)**.

To summarize, the experimental studies and the clinical experiences indicate that during moderate cerebral hypermetabolism (arousal, extubation) is associated with an insignificant increase in LP ratio and modest, almost parallel increases in lactate and pyruvate During very pronounced hypermetabolism (epileptic seizures) LP ratio and lactate increases to very high levels accompanied by a moderate increase in pyruvate. After prolonged experimentally induced seizures a moderate decrease in intracerebral glucose occurs. A decrease in glucose has been described during prolonged seizures in patients. The time course of the changes in lactate, pyruvate and LP ratio as well as the simultaneous expected changes in CBF/PbtO_2_ are schematically illustrated in Figures 3C,D.

## Ischemia, mitochondrial dysfunction and hypoxia

The concept of ischemia originates from the Greek words *ischein* (to restrain) and *haima* (blood) and is commonly defined as insufficient organ perfusion irrespective of the cause. Under experimental and clinical conditions ischemia will cause different biochemical consequences depending on the degree of CBF restriction. The changes after a momentary, complete interruption of CBF has been studied extensively in animal experiments. In this situation a severe perturbation of energy metabolism is measured within seconds as a marked increase in LP ratio. After a few min glucose and pyruvate are completely depleted and lactate has increased to a maximum level (approximately 12 mmol/L) (44–46). When the substrate has been consumed a prolongation of complete ischemia will not result in a further increase in lactate.

As the LP ratio reflects cytoplasmatic redox-state, an increased ratio does not necessarily indicate cerebral ischemia. Accordingly, in patients treated for severe traumatic brain lesions it has repeatedly been described that increased LP ratio was not explained by a decrease in CBF (62, 63). An increase in LP ratio is also expected in mitochondrial dysfunction.

The biochemical pattern of mitochondrial dysfunction obtained utilizing microdialysis has been described after intracerebral infusion of cyanide in the pig (48). Mitochondrial dysfunction results in an increased LP ratio at normal or elevated CBF (PbtO_2_) level and is characterized by a marked increase in cerebral lactate at a normal or elevated pyruvate level (Figure 3B). The metabolic pattern is quite different from pronounced cerebral ischemia, which is characterized by simultaneous decreases in intracerebral pyruvate and CBF (PbtO_2_) (Figure 3A).

**Figure 4:**
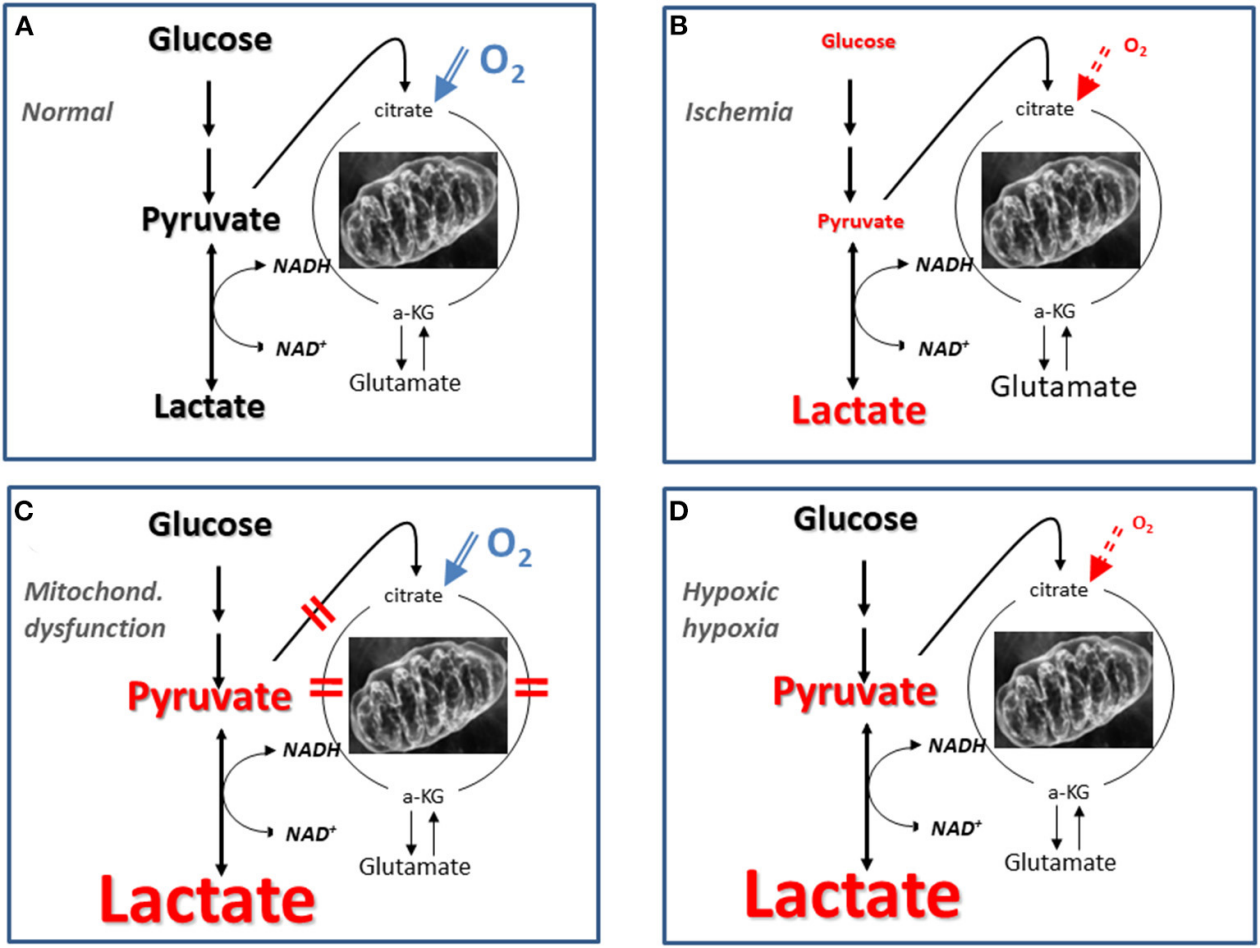
Schematic illustration of biochemical patterns obtained utilizing intracerebral microdialysis under normal conditions **(A)**, during cerebral ischemia **(B)**, mitochondrial dysfunction **(C)**, and hypoxic hypoxia **(D)**. Mitochondrial dysfunction may be caused by lesions at various steps in the citric acid cycle as indicated by the red bars in **(C)**. The font size reflects the level of the various variables in three conditions compared to their normal reference concentrations.

It should be underlined that the pattern of LP ratio, lactate and pyruvate described for mitochondrial dysfunction (Figure 3B) is not unique for this condition. Figure 4 gives a schematic illustration of the metabolic pattern during normal conditions (A), ischemia (B), mitochondrial dysfunction (C) and hypoxic hypoxia (D). As illustrated in Figure 4D a metabolic pattern similar to mitochondrial dysfunction would be obtained during a selective decrease in arterial oxygen supply (hypoxic hypoxia) (47). Under clinical conditions this fact will hardly cause confusion as arterial oxygen level is routinely monitored. However, as discussed previously, a gradual decrease in CBF will result in insufficient cerebral tissue oxygenation before substrate delivery is compromised. As discussed later, this metabolic pattern is transient. When a lasting pattern is observed during clinical conditions it should be interpreted as mitochondrial dysfunction (64).

## Cerebral energy metabolism in transient ischemia

Transient cerebral ischemia is common in critical care. The episodes may occur as a single, focal (e.g., cerebral embolism), or global (e.g., hemorrhagic shock, cardiac arrest) event or repeatedly in various severe conditions during neurocritical care. Therefore, to interpret biochemical data obtained during cerebral microdialysis correctly, it is necessary to consider the corresponding data obtained under controlled experimental conditions.

### Transient ischemia studied in brain tissue homogenates

The biochemical changes occurring after transient cerebral ischemia have been extensively investigated utilizing brain tissue homogenates in animal experiments (1, 65–67). In these studies, an immediate interruption of CBF was attained by a momentary increase in intracranial pressure (ICP) above arterial blood pressure. Cerebral energy metabolism was evaluated during 5–30 min of complete ischemia and a following momentary, adequate recirculation. After 30 min of complete ischemia and a 90 min period of recirculation, biochemical analyses showed restitution of cerebral cortex concentrations of organic phosphates, glycolytic metabolites, LP ratio, citric acid cycle intermediates and associated amino acids (67). Altogether, the experiments demonstrated extensive normalization of mitochondria1 metabolism after prolonged, complete cerebral ischemia.

Under identical experimental conditions, cortical mitochondria were isolated and studied *in vitro*. After 30 min of ischemia there was a decrease in respiratory control ratio (RCR), in state 3 respiratory activity and maximal phosphorylation rate. However, after recirculation the mitochondria showed extensive functional recovery with normalization of RCR, as well as of state 3 and maximal phosphorylation rates (68). Accordingly, mitochondrial function and cerebral aerobic metabolism would be expected to recover after 30 min of ischemia provided optimal recirculation can be achieved. If optimal recirculation could be attained under clinical conditions, a similar biochemical pattern would be expected.

A second experimental model was developed to imitate the clinical conditions during transient cerebral ischemia in patients. In this model cortical blood flow was not completely interrupted but reduced to about 5 % of control (69). Following 30 min of severe, incomplete ischemia energy metabolism did not recover and 90 min after start of recirculation massive increases in lactate, LP ratio and pyruvate were obtained (70).

Deficient recirculation has been suggested as a factor limiting restitution following transient cerebral ischemia. Insufficient microcirculation may be caused by a combination of pathophysiological mechanisms such as swelling of endothelial and perivascular cells, intravascular aggregation of blood corpuscles, and increased blood viscosity (71–74). However, in the experimental model discussed quantitative measurements of CBF did not support this hypothesis (75) and the biochemical pattern did not indicate that insufficient recirculation was the primary cause of the biochemical collapse (70). *In vitro* studies on isolated mitochondria showed remaining mitochondrial dysfunction after recirculation of incomplete ischemia in contrast to following complete ischemia (68). In severe, incomplete cerebral ischemia a continuous supply of glucose at insufficient tissue oxygenation results in excessive accumulation of lactate. Many subsequent experimental studies have shown that excessive lactic acidosis during profound cerebral ischemia entails extensive mitochondrial and tissue damage (76–78).

To sum up, animal experiments have documented that mitochondrial function and cerebral aerobic energy metabolism may recover to a large extent after prolonged complete ischemia (30 min) provided adequate recirculation is accomplished. The observation is of importance for restitution e.g., after cardiac arrest and resuscitation. This aspect is discussed in section 8.3. (Microdialysis during resuscitation after out of hospital cardiac arrest).

### Intracerebral microdialysis in transient experimental ischemia

**Figure 5:**
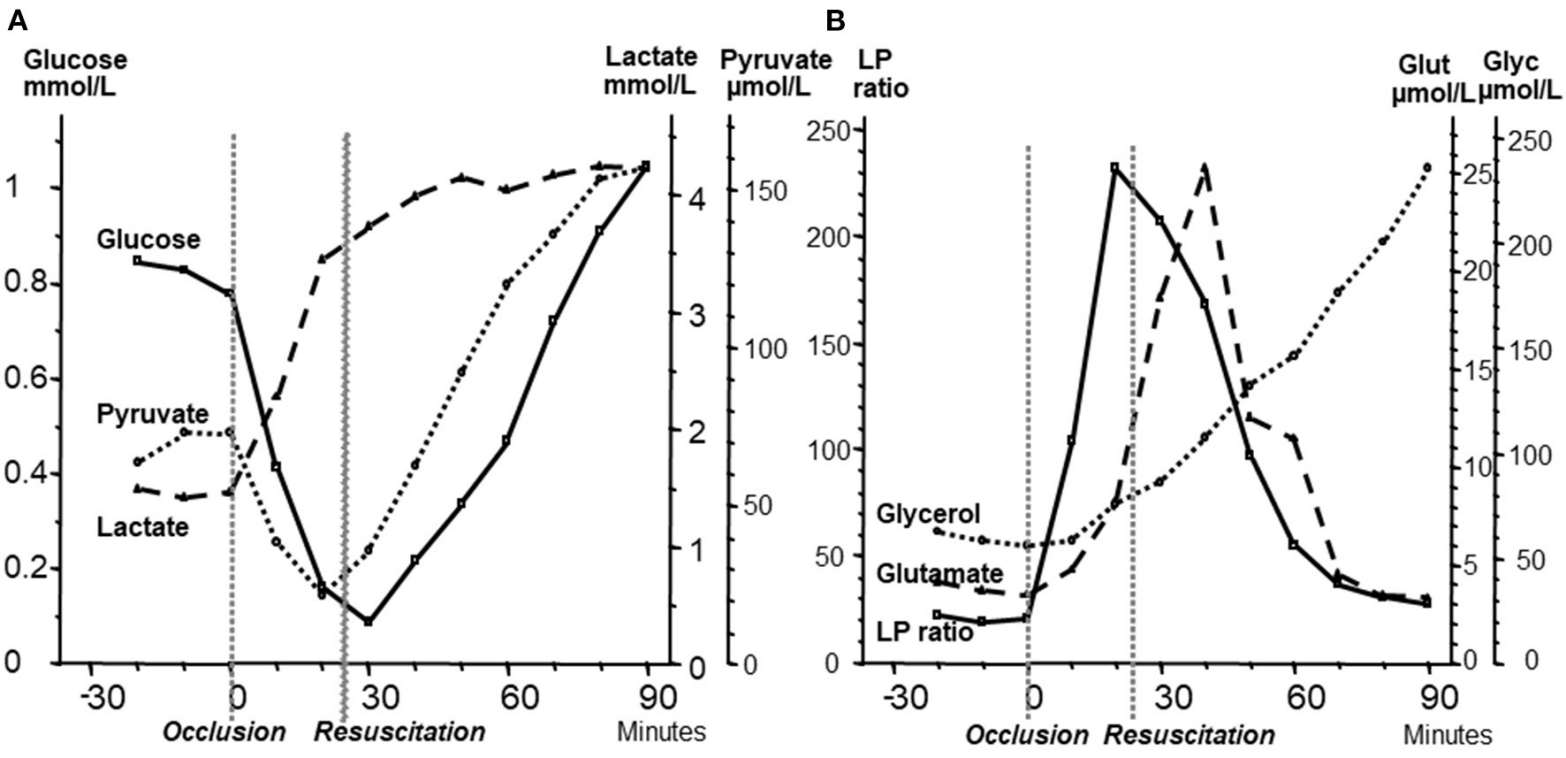
**(A):** Changes in fetal intracerebral levels of glucose, pyruvate and lactate during cardiac arrest due to umbilical cord occlusion and after resuscitation. Umbilical occlusion and start of resuscitation are indicated in the figure. **(B):** Changes in intracerebral levels of lactate/pyruvate (LP) ratio, glutamate, and glycerol after umbilical cord occlusion and after resuscitation. Data (Mean levels) from (79).

For the interpretation of clinical microdialysis data, it is necessary to compare the results obtained from studies utilizing brain homogenates with those observed during transient experimental ischemia and intracerebral microdialysis. For this purpose, transient brain ischemia was induced in fetal lambs *in utero* by occlusion of the umbilical cord followed by resuscitation after cardiac arrest (79). The microdialysis technique was identical to that used during clinical conditions, but the perfusion rate was increased (1.0 μl/min) to allow frequent sampling. Induction of ischemia caused an almost instantaneous increase in the LP ratio followed by an increase of the glutamate level (Figure 5B). Glucose, pyruvate, and glutamate rapidly recovered after resuscitation, but the levels of lactate and glycerol continued to be elevated. The high LP ratio rapidly decreased to close to normal within 30 min but remained slightly above the pre-ischemic level (Figures 5A,B). In a recent study of transient cerebral ischemia in awake rats, a similar biochemical pattern (a remaining increase in lactate at a normal or slightly increased pyruvate concentration) indicating persisting mitochondrial dysfunction was observed (80) and resulted in various attempts to treat the condition and to document the treatment effect (80–82).

These microdialysis data in these experimental studies correspond to those obtained from analyses of brain homogenates and are of fundamental importance for the interpretation of the changes observed during clinical microdialysis. Accordingly, the LP ratio would be expected to increase immediately when delivery of oxygen is insufficient and rapidly return to nearly normal levels upon sufficient re-oxygenation (79). The lactate level is expected to increase rapidly during ischemia and remain elevated when circulation is restituted. Glycerol, the indicator of degradation of cellular membranes, would be expected to increase relatively slowly during energy failure and remain elevated for a period when energy metabolism is normalized. The interstitial glucose level reflects the balance between delivery from the blood capillaries and cellular uptake. These variables have also been measured and interpreted in a number of experimental studies of resuscitation after cardiac arrest and involves the influence of cerebral perfusion pressure, cerebral oxygenation, targeted temperature, adrenaline infusion and secondary deleterious events (83–88). Interpretation of the biochemical patterns observed under clinical microdialysis are based on similar principles.

### Intracerebral microdialysis in transient clinical ischemia

In transient clinical ischemia it is important to interpret the perturbation of cerebral energy metabolism remaining after recirculation correctly: are the observed changes due to continuing ischemia or caused by disturbed aerobic energy metabolism (mitochondrial dysfunction)? We have previously discussed the possibility to separate ischemia from mitochondrial dysfunction from the pattern of the LP ratio in relation to the levels of lactate and pyruvate. These observations were obtained under experimental conditions and may be compared with clinical experiences in patients after recirculation of middle cerebral artery (MCA) infarcts, subarachnoid hemorrhage (SAH), bacterial meningitis and in patients treated for severe brain trauma.

**Figure 6:**
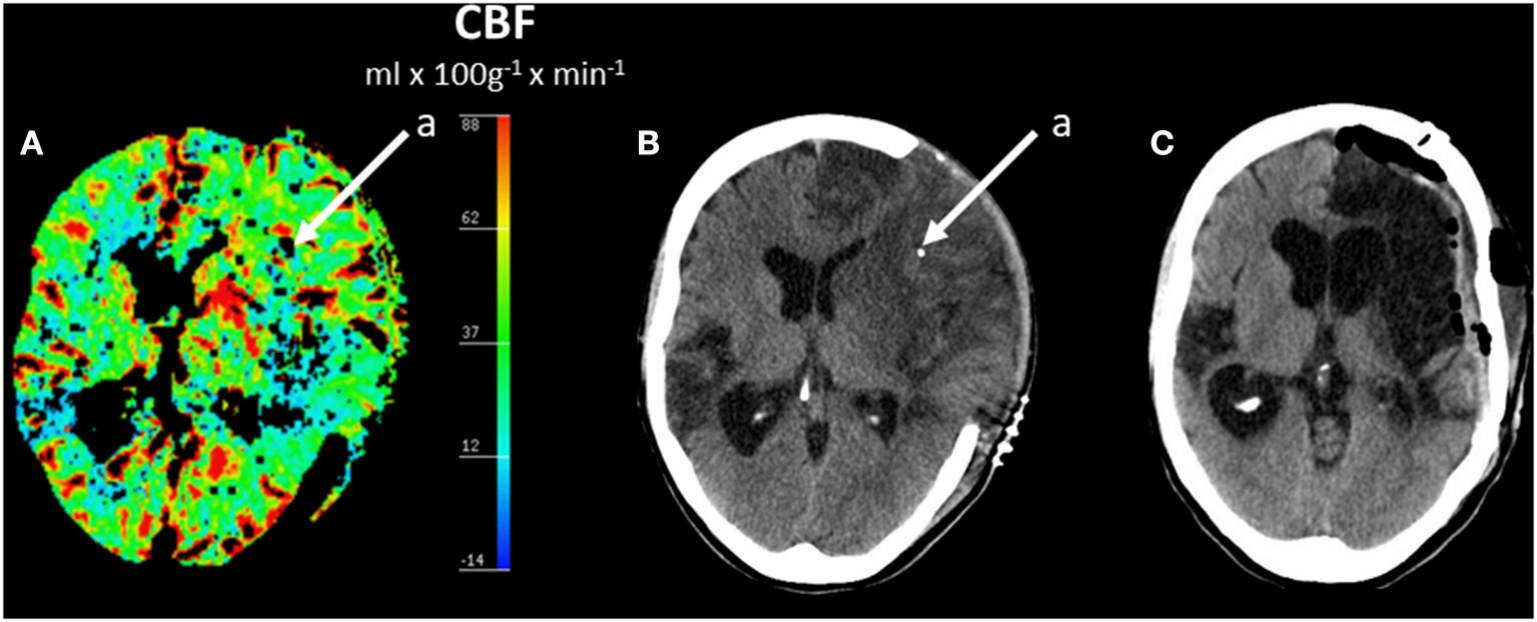
Regional cerebral blood flow (CBF) after hemicraniectomy in a patient with malignant brain edema after middle cerebral artery (MCA) infarct **(A)** and corresponding acute CT scan **(B)** and CT scan after 6 months **(C)**. **(A,B)** Shows the position of one intracerebral microdialysis catheter (a) in the infarcted hemisphere. **(C)** Shows the CT scan 6 months later after replacement of the skull bone. Data from (89).

**Figure 7:**
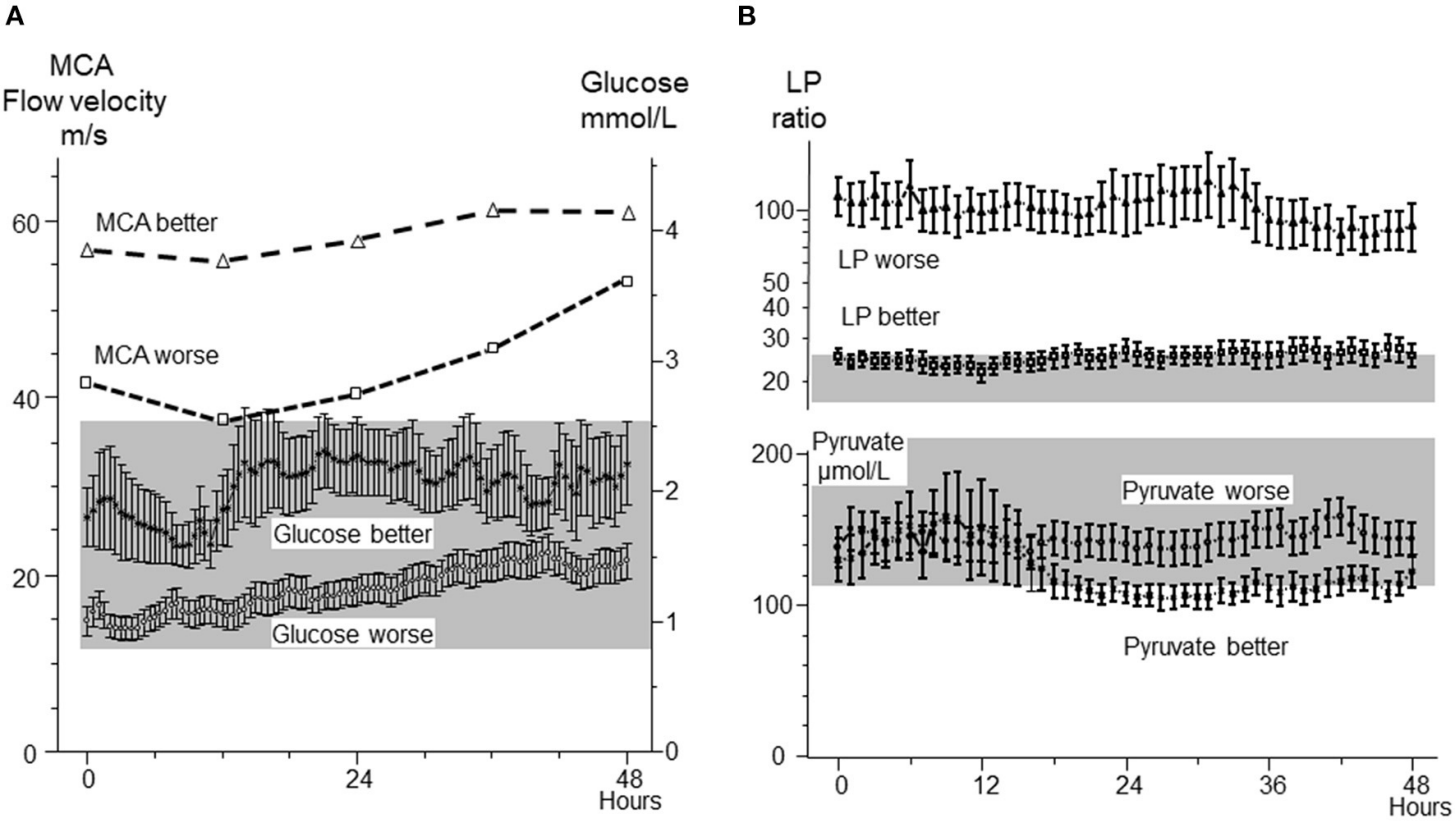
**(A):** Middle cerebral artery (MCA) blood-flow velocities in the infarcted (worse) and non-infarcted (better) hemisphere and cerebral interstitial glucose concentration in infarcted (worse) and non-infarcted (better) hemisphere following hemicraniectomy. **(B):** Cerebral interstitial levels of lactate/pyruvate (LP) ratio and pyruvate in the infarcted (worse) and non-infarcted hemisphere (better) after hemicraniectomy. The normal ranges for intracerebral glucose and pyruvate (Mean ± 2 S.D.) (41) are indicated by the gray areas. Biochemical data from (89, 90) are shown as Mean ± SEM.

The biochemical patterns observed following severe cerebral ischemia and recirculation is illustrated by the observations after reperfusion in large MCA infarcts. A consecutive series of 44 patients with MCA infarcts and malignant brain swelling were treated with hemicraniectomy. Microdialysis catheters were inserted into the infarcted and the contralateral hemispheres (89, 90). Figure 6 illustrates regional CBF and the position of one microdialysis catheter after recirculation and hemicraniectomy. Comparisons between infarcted (worse) and non-infarcted hemisphere (better) are shown for MCA blood flow velocity and glucose (Figure 7A), LP ratio and pyruvate (Figure 7B). In the infarcted hemisphere the variables exhibited a prolonged increase in LP ratio at a normal or slightly elevated concentration of pyruvate. The pattern corresponds to that observed during induced mitochondrial dysfunction (48). In the infarcted hemisphere, intracerebral glucose level was close to normal while lactate and glutamate remained high. Similar biochemical indications of mitochondrial dysfunction have been described in patients with SAH and severe bacterial meningitis (91–93).

**Figure 8:**
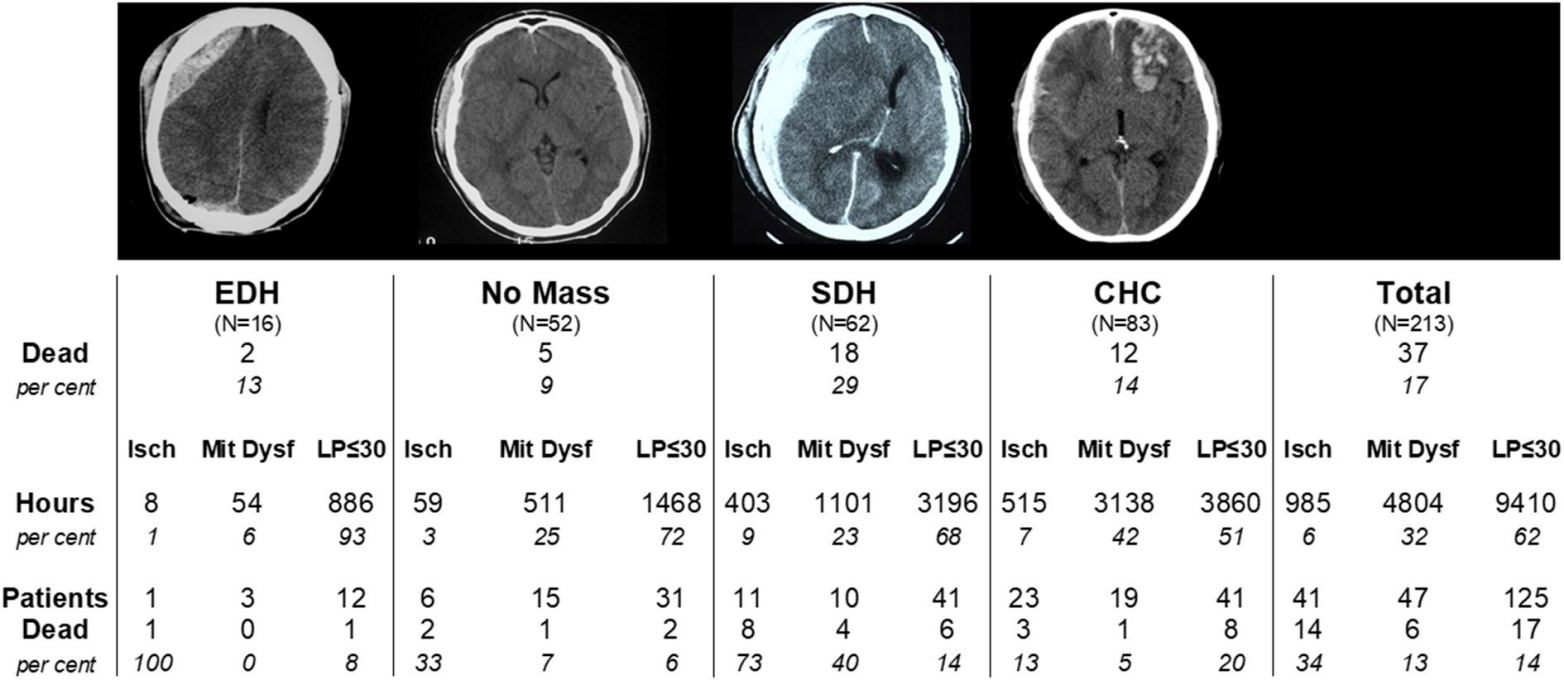
Total mortality and mortality in four diagnostic groups of severe traumatic brain lesions in 213 patients: Extradural hematoma (EDH); Acute subdural hematoma (SDH); Focal cerebral hemorrhagic contusion (CHC); No Mass denotes patients not treated with surgical evacuation. Mortality is also shown separately for the biochemical subgroups defined as Ischemia (Isch), Mitochondrial Dysfunction (Mit Dysf) and normal aerobic metabolism (LP-ratio ≤ 30) within each diagnostic group. Data from (64).

As previously mentioned, a discrepancy between CBF and cerebral energy metabolism was described in patients treated for severe brain trauma (62, 63). In a large study including 213 patients with severe brain trauma monitored with altogether 342 intracerebral microdialysis catheters the relations between clinical outcome (mortality), type of lesion (extradural hematoma, no mass lesion, acute subdural hematoma, focal cerebral hemorrhagic contusion) and perturbation of cerebral energy metabolism (ischemia vs. mitochondrial dysfunction) were explored (64). During neurocritical care a biochemical pattern indicating mitochondrial dysfunction was more frequent than a pattern of cerebral ischemia (32 and 6% of monitored time, respectively) but mortality was more common in patients with an ischemic pattern (34 vs. 13%). A detailed account of the results is given in Figure 8.

In summary, transient cerebral ischemia is a common clinical problem and bedside interpretation of the metabolic patterns obtained may be used to direct therapy. The interpretation should be based on knowledge of the vast amount of experimental data from whole brain analyses and their correlates obtained utilizing microdialysis. During the interpretation the biochemical patterns observed in various pathophysiological conditions must be considered: e.g., ischemia, mitochondrial dysfunction, hypoxic hypoxia, arousal, non-convulsive epileptic activity and epileptic seizures.

## Microdialysis of cerebral venous blood—experimental

**Figure 9:**
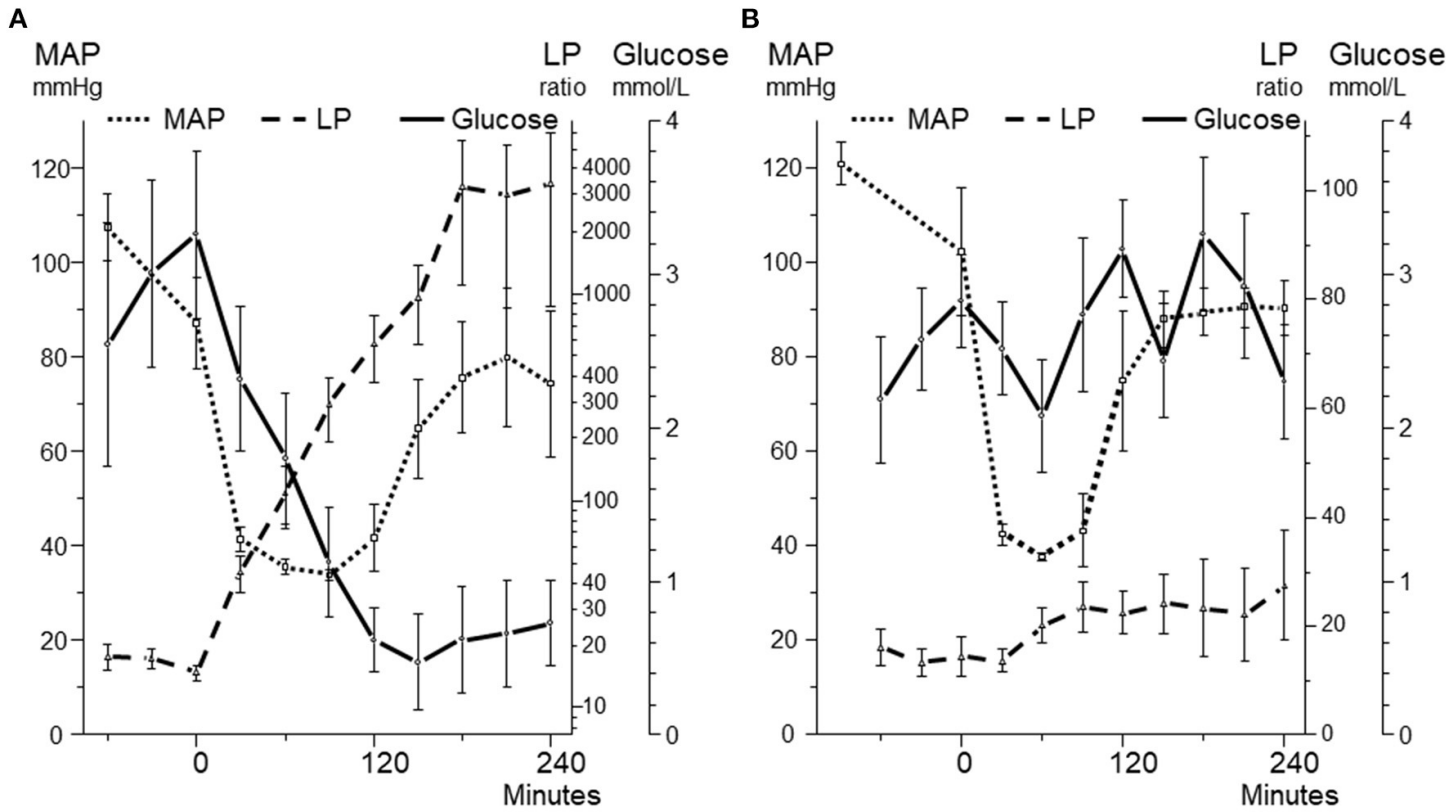
Mean arterial blood pressure (MAP) and intracerebral levels of glucose and lactate/pyruvate (LP) ratio during 240 min before, during and after a 90 min period **(A)** and a 60 min period **(B)** of hemorrhagic shock. In both panels MAP decreased to 40 mmHg. Panel A illustrates the biochemical pattern during severe cerebral ischemia. Panel B illustrates a pattern when intracerebral glucose an LP ratio are relatively unaffected during a similar but shorter decrease in MAP. Data from (94, 95) are shown as Mean ± SEM.

**Figure 10:**
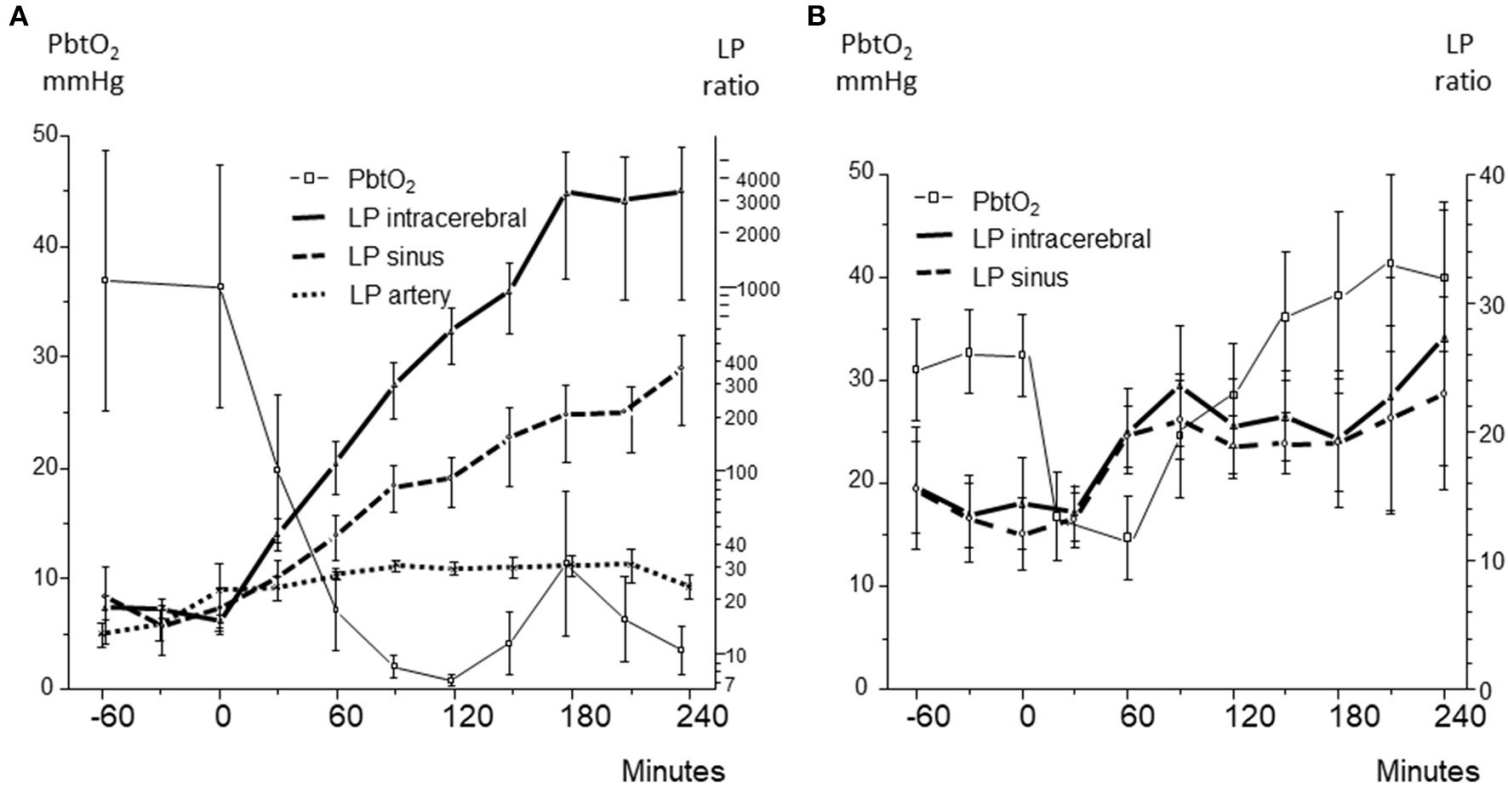
**(A):** Brain tissue oxygen tension (PbtO_2_), and lactate/pyruvate (LP) ratio obtained in brain tissue (intracerebral), superior sagittal sinus (sinus), and femoral artery (artery) during severe, irreversible ischemia after induced hemorrhagic shock. **(B):** Brain tissue oxygen tension (PbtO_2_), and lactate/pyruvate (LP) ratio obtained in brain tissue (intracerebral) and superior sagittal sinus (sinus) during reversible hemorrhagic shock. Data from (94, 95) are shown as Mean ± SEM.

An experimental model of global cerebral ischemia during hemorrhagic shock in the pig was used to explore whether cerebral energy state might be evaluated from microdialysis of the draining cerebral venous blood (94). In the first series of experiments a mean arterial blood pressure (MAP) of 40 mmHg was below the autoregulatory capacity as illustrated by a rapid intracerebral increase in LP ratio and decreases in glucose (Figure 9A) as well as a brain tissue oxygenation (PbtO_2_) (Figure 10A) (94). After reinfusion of blood a transient increase in PbtO_2_ was observed (Figure 10A) but, due to progressive brain swelling, energy metabolism did not recover. Figure 10A illustrates the simultaneous changes in LP ratio in the intracerebral compartment and in venous (superior sagittal sinus) and arterial (femoral artery) blood.

During severe global brain ischemia microdialysis of the venous drainage gave qualitative information of cerebral energy metabolism that could be separated from the perturbation of energy metabolism in the rest of the body. In accordance with the biochemical pattern in ischemia (Figures 3A, 5B) pyruvate concentration decreased in the intracerebral compartment as well as in the sagittal sinus.

In the following series with a shorter period of hemorrhagic shock, the animals initially tolerated a decrease of MAP to 40 mmHg (Figure 9B) (95). A moderate increase in intracerebral LP ratio at an essentially unaffected glucose level was observed (Figure 9B). The less pronounced and shorter decrease in PbtO_2_ was reflected in modest increases in intracerebral as well as venous LP ratio (Figure 10B) and no decrease in pyruvate level in the sagittal sinus was obtained.

In summary, these experimental studies demonstrated that during severe, global brain ischemia semiquantitative information of cerebral energy state could be obtained from microdialysis of the draining cerebral venous blood. In particular, the technique might be of interest in severe, widely spread cerebral ischemia when it is difficult or impossible to insert an intracerebral microdialysis catheter under clinical conditions (e.g., during resuscitation after cardiac arrest).

## Microdialysis of cerebral venous blood—clinical

The biochemical information obtained from microdialysis of the draining cerebral blood has been evaluated in three clinical situations: during neurocritical care in patients with SAH, during cardiopulmonary bypass (CPB) in open heart surgery, and during intensive care in patients resuscitated after out of hospital cardiac arrest (OHCA). In all three groups of patients, the intravenous microdialysis catheter was placed in a retrograde direction in the jugular bulb. To prevent clot formation around the microdialysis membranes dalteparin sodium (25 IU/mL) was added to the perfusion fluid of all intravenous and intraarterial catheters in accordance with the recommendations from the manufacturer (M Dialysis, Stockholm, Sweden).

### Microdialysis of cerebral venous blood in subarachnoid hemorrhage

This observational study aimed to explore the feasibility of jugular bulb microdialysis (JBMD) in SAH and describe the output characteristics in relation to conventional multimodal monitoring. In particular, it would be of interest to clarify whether episodes of severe cerebral ischemia revealed by intracerebral microdialysis was reflected by JBMD.

Twelve patients treated for aneurysmal SAH were included in the study and monitored for a mean period of 4.2 ± 2.6 days (96). Monitoring was initiated within 48 h of admission after all aneurysms had been secured by either endovascular coiling or open surgical clipping. All survivors presented with a favorable outcome at the 6-month outpatient examination.

The patients were monitored using a non-dominant frontal cerebral microdialysis catheter (70 MD Bolt Catheter, M Dialysis AB, Stockholm) with an ICP and brain oxygen tension (PbtO_2_) probe in a double lumen bolt (PTO2L, Raumedic^®^, Helmbrechts). The JBMD catheter (67 IV MD Catheter, M Dialysis AB, Stockholm) was inserted 5 cm above the clavicular bone through a 16 G *i.v*. cannula. Correct placement of cerebral and jugular bulb catheters (gold tip) was confirmed through regular control computed tomography (CT) imaging.

The study concluded that continuous microdialysis monitoring of the cerebral drainage in the jugular bulb was feasible and safe in patients treated for SAH. With the exception of glucose, there were no significant correlations between intracerebral microdialysis and JBMD at cohort level. The limited number of patients included in this feasibility study precluded a definite answer to the question whether severe cerebral ischemia would be reflected in JBMD. Only two patients (pat. 3 and pat. 9) exhibited an intracerebral biochemical pattern of ischemia.

In pat. 9 intracerebral microdialysis (LP ratio 554, pyruvate 6 μmol/l, glucose 0.3 mmol/l, glutamate 424 μmol/L) was not reflected in JBMD. However, the pattern of metabolites in JBMD documented that the catheter was outside the blood vessel (glutamate 18 μmol/L) and the catheter could not be visualized on CT-scanning.

In pat. 3 intracerebral data indicated severe ischemia (LP ratio 140, pyruvate 40 μmol/L, glucose 0.02 mmol/L, glutamate 109 μmol/L) but all variables were within normal limits in JBMD. In this patient CT showed a minimal hematoma without clinical significance surrounding the intracerebral catheter and the 6-months follow up was favorable.

The latter patient demonstrated that a pathological biochemical pattern obtained during intracerebral microdialysis may be due to a very local lesion without clinical significance. As in most neurosurgical conditions perturbation of energy metabolism is initially local, an early warning of deterioration will be obtained provided the microdialysis catheter is positioned in the penumbra of the focal lesion (Figure 1) (27). The JBMD technique would not be expected to give this information until a large part of the brain has been involved. Accordingly, the JBMD technique might be expected to give information of clinical importance only in patients who have suffered from permanent or transient global cerebral ischemia.

Another microdialysis technique for evaluation of global cerebral energy metabolism during neurocritical care has been tested (97). In a limited group of patients with traumatic brain injuries a microdialysis catheter was positioned in a ventricular catheter used for continuous CSF drainage. This preliminary study does not show whether clinically useful information may be obtained. In comparison with the JBMD technique two disadvantages are noted: (1) the time delay from the intracerebral compartment of interest to the microdialysis catheter is unknown and probably variable; (2) the technique can only be used after insertion of a ventricular drain which is usually difficult or impossible during general intensive care (e.g., cardiopulmonary surgery, resuscitation after cardiac arrest).

### Microdialysis during cardiopulmonary bypass

Despite considerable progress in surgical CPB and anesthetic techniques brain damage remains an important complication of cardiac surgery (98, 99). In a feasibility study 10 patients undergoing primary, non–emergency coronary artery bypass grafting were blindly randomized to low or high MAP (43). Calculated laminar flow during CPB was identical in the two groups. The difference in MAP between groups was significant: low MAP 44 (IQR, 41–49) mmHg vs. high MAP 65 (IQR 60–76) mmHg (43). One microdialysis catheter (70, M Dialysis AB, Stockholm, Sweden) was placed in the jugular bulb and a second identical catheter was inserted into one brachial artery. The objective of the arterial catheter was to enable differentiation between cerebral metabolic perturbation from biochemical effects in the rest of the body. Both catheters were inserted through a peripheral intravenous 17-G cannula using ultrasound guidance. The positioning of the catheter in the jugular bulb above the inlet of the common facial vein was verified on lateral neck radiograph. Perfusion and analysis were performed as previously described (section 7.1). In all patients regional cerebral oxygen saturation was monitored using bifrontal NIRS (Somanetics INVOS Cerebral Oximeter system) preoperatively, intraoperatively and for 2 h post operatively.

**Figure 11:**
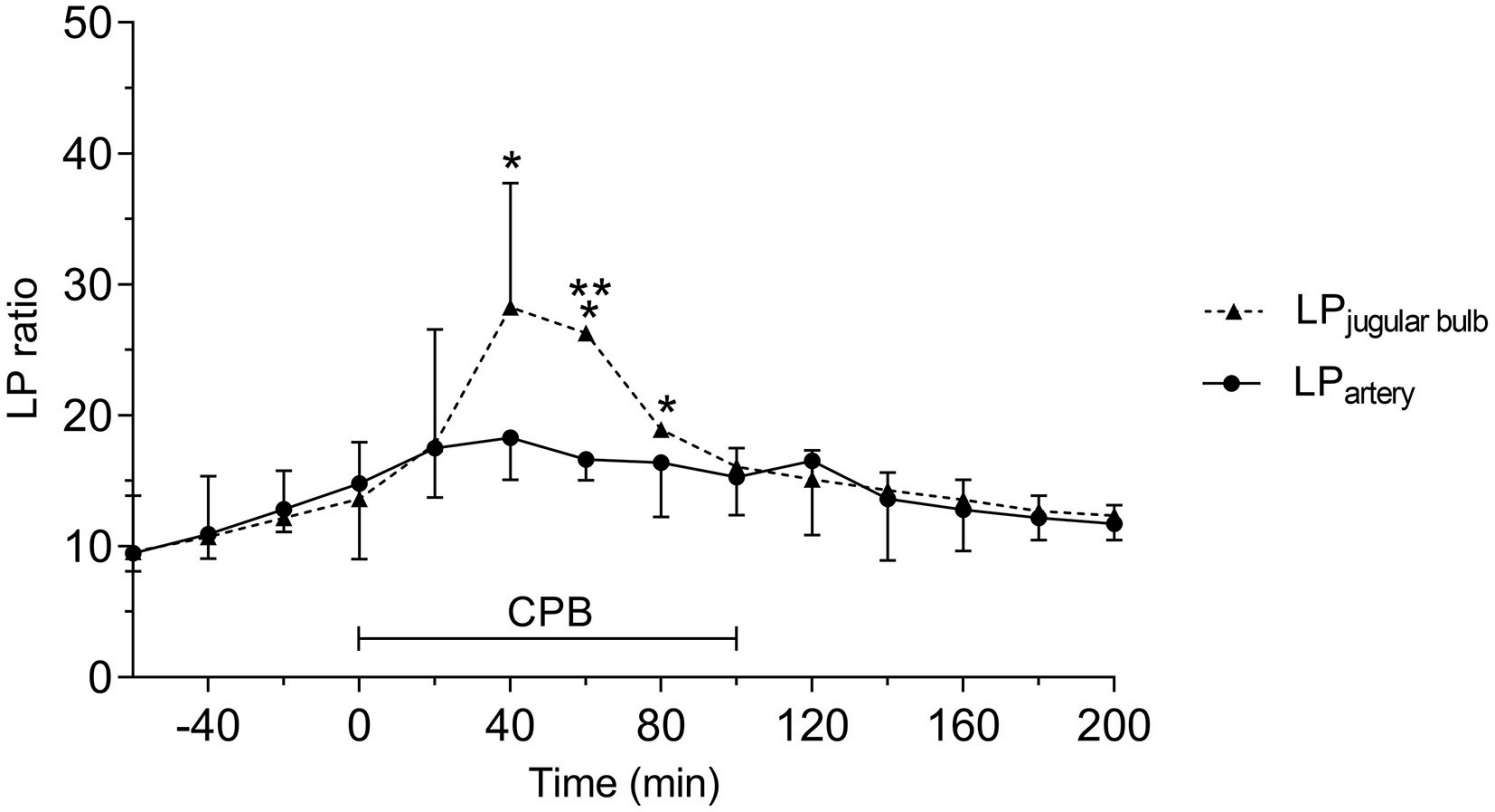
Acute effects of cardiopulmonary bypass (CPB) on jugular bulb lactate/pyruvate (LP) ratio and peripheral artery LP ratio. *Significant difference from baseline. **Significant difference between corresponding datapoints by one-way analysis of variance and corrected for multiple comparisons using the Bonferroni test (α = 0.006). Values are median and error with inter quartile range (*n* = 10). Figure by permission from (43).

The LP ratio monitored simultaneously from jugular bulb and intraarterial microdialysis are illustrated in Figure 11. During CPB the peak LP ratio was significantly higher in JBMD documenting that the LP ratio obtained in the jugular bulb was a reflection of cerebral energy metabolism. The increase in intravenous LP ratio above normal level (43) was interpreted as indicating compromised cerebral oxidative metabolism due to a decrease in CBF when MAP was below the normal range of pressure autoregulation. (60–160 mmHg) (100).

**Figure 12:**
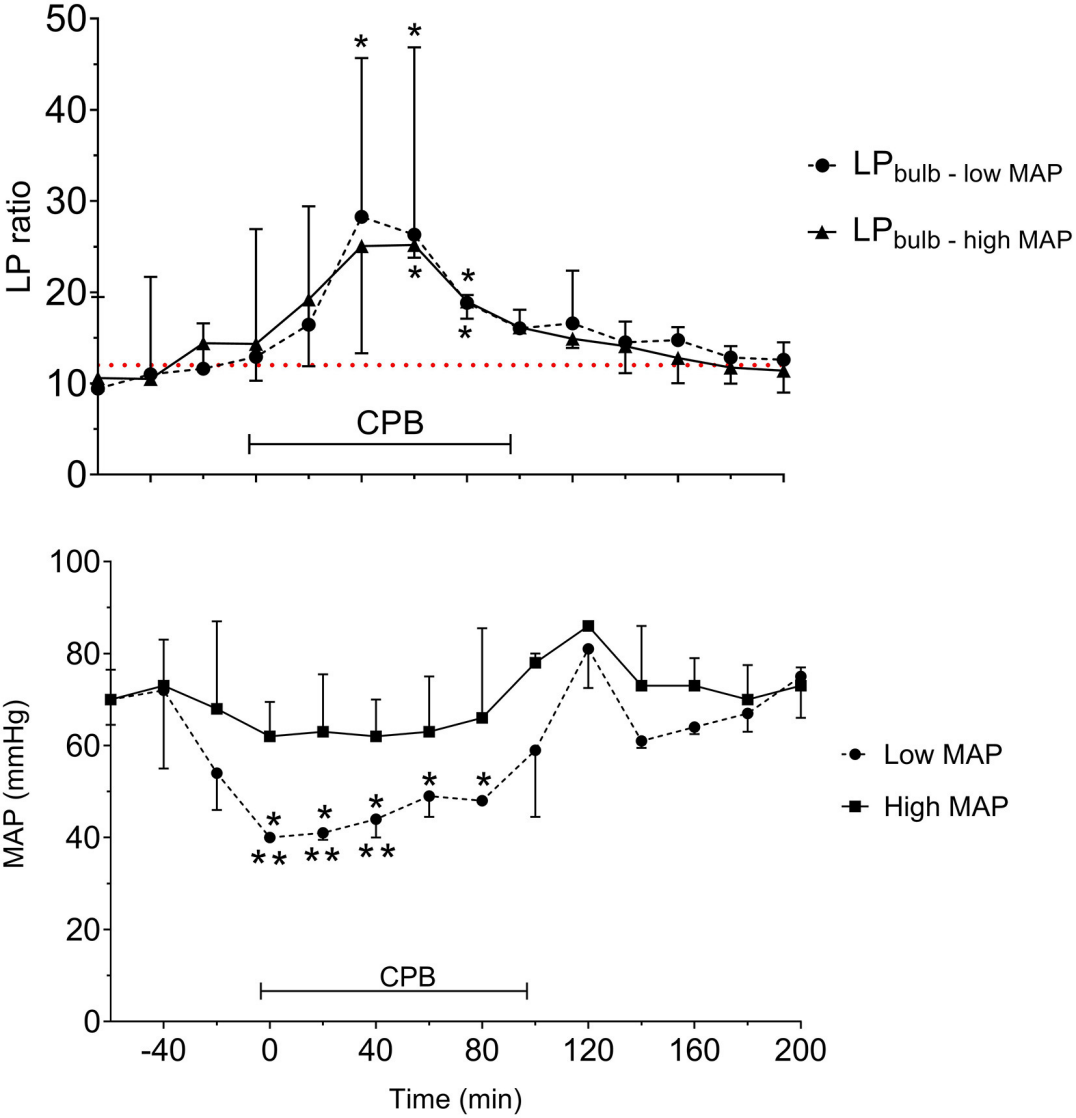
Acute effects of cardiopulmonary bypass (CPB) and two levels of mean arterial blood pressure (MAP) on lactate/pyruvate (LP) ratio obtained from a microdialysis catheter placed in the jugular bulb (upper panel) and the simultaneous MAP levels (lower panel). Data are shown as median (IQR). The interrupted line (upper panel) indicates reference level (Mean) for LP ratio in human jugular venous blood (43). *Significant difference from baseline. **Significant difference between corresponding datapoints by one-way analysis of variance and corrected for multiple comparisons using the Bonferroni test (α = 0.006). Values are median and error with inter quartile range (*n* = 10). Figures by permission from (43).

The LP ratios obtained from JBMD in the two MAP groups are shown in Figure 12 (upper panel). The difference in MAP between the two groups was significant (Figure 12, lower panel). After initiating CPB, the mean LP ratio increased significantly by 160% (low MAP) and 130% (high MAP). In both groups, the mean peak LP ratio also increased significantly. However, although low-MAP patients had a tendency to have higher LP ratios (Figure 12, upper panel) the difference between groups was not statistically significant. In both groups, LP ratio returned to baseline after CPB. In spite of the obvious increase in LP ratio during CPB no cerebral desaturations (decrease in rSO2 < 20% from baseline) were observed by NIRS in either group.

In summary, the study documented that it is feasible to place a microdialysis catheter in the jugular bulb during CBP and open-heart surgery. The LP ratio of cerebral venous blood increased significantly during CPB, indicating compromised cerebral oxidative metabolism, and was correlated to the decrease in MAP. In this limited number of patients, there was no significant difference between low-and high-MAP groups regarding venous outflow LP ratio during CPB. Low MAP patients tended to have higher LP ratios but conventional rSO_2_ monitoring utilizing NIRS did not show a corresponding decrease in cerebral oxygenation. As some patients exhibited decreased cognitive functions after CPB, the study indicated that an increase in jugular venous LP ratio might be a sensitive indicator of impending cerebral damage.

### Microdialysis during resuscitation after out of hospital cardiac arrest

Survival rates around 50% are reported in comatose patients resuscitated after out-of-hospital cardiac arrest OHCA (101–103). The mortality during intensive care is essentially due to the primary hypoxic-ischemic cerebral insult followed by secondary brain injuries. These include delayed cerebral hypoperfusion and impaired microcirculation as well as ischemia–reperfusion injury (104, 105). As secondary injury is a significant determinant of neurologic outcome alleviation of its deleterious effects is in focus during post-cardiac arrest management (106). However, the possibilities of monitoring cerebral energy metabolism have so far been very limited. Intracerebral microdialysis is an established technique in neurocritial care but can hardly be used as a routine technique in this group of patients. In this situation JBMD might offer a new possibility.

A feasibility study was designed to investigate if bedside JBMD reflected secondary deterioration of cerebral energy metabolism after OHCA and whether it might be implemented as a clinical tool for early evaluation of prognosis and individualization of treatment (107). To document whether JBMD reflected intracerebral energy state the primary objective of the study was to compare time-averaged means of lactate, pyruvate and LP ratio (intervals of 12 h) of the jugular venous and the arterial blood during post-resuscitation care. Secondary objectives of clinical interest were to compare (a) neuro-metabolic patterns between patients with unfavorable and favorable neurological outcome (b) total duration of cerebral desaturation and clinical outcome.

Eighteen unconscious patients with sustained return of spontaneous circulation (ROSC) after OHCA were included in the study (107). JBM and near-infrared spectroscopy monitoring were the sole modifications from international clinical treatment guidelines for comatose OHCA patients. Immediate angiography and percutaneous coronary intervention, when indicated, was performed in all resuscitated patients. Targeted temperature management (TTM) was commenced at the time of ICU admission targeting 36.0°C for 24 h followed by controlled rewarming at a rate of 0.5°C/h. A mean arterial pressure (MAP) > 65 mmHg was targeted. Mechanical ventilation was adjusted to achieve normocapnia (PaCO_2_ of 4.5–6.0 kPa) and oxygenation was maintained in the range of 13–14 kPa. Blood-glucose level was strictly maintained between 6 and 10 mmol/l.

JBMD was initiated after ICU admission (approximately 300 min after ROSC) and continued for 96 h or until arousal. Intravenous MD catheters (67 IV, M Dialysis AB, Stockholm, Sweden) were inserted in one jugular vein and one peripheral artery and perfused and analyzed as previously described (section 8.1). The definition of normal levels of the studied variables in human jugular vein blood was based on JBM reference values obtained in anesthetized patients undergoing elective cardiac bypass surgery (43). The correct positioning of the jugular bulb catheter tip was confirmed on cranial CT scanning.

**Figure 13:**
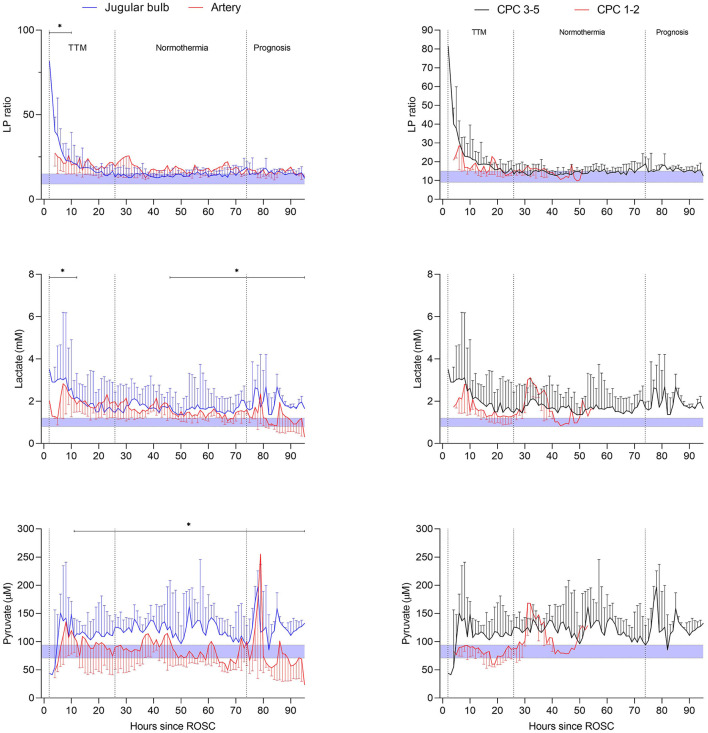
Left panels: microdialysis variables (LP ratio, lactate, pyruvate) of the jugular venous and arterial blood during post-resuscitation care after out of hospital cardiac arrest in patients with unfavorable outcome. Right panels: jugular bulb microdialysis variables (LP ratio, lactate, pyruvate) during post-resuscitation care in patients with unfavorable outcome (CPC 3–5) compared with patients with favorable outcome (CPC 1–2). The period of targeted temperature management (TTM) is indicated in the figures. The difference between time-averaged means (in intervals of 12 h) of LP ratio, lactate, pyruvate, glycerol and glutamate of the jugular venous and the arterial blood was significant during post-resuscitation care (*p < 0.02) when using mixed effects models. Data are shown as median (IQR). Shaded areas represent normal reference levels for the variables during jugular bulb microdialysis (43). Figures by permission from (107).

The changes over time in arterial and jugular microdialysis for patients in the poor outcome (CPC 3–5) group ratio is compared in Figure 13 (left panels) regarding lactate and pyruvate as well as the calculated LP ratio. The difference between time-averaged means of LP ratio, lactate and pyruvate were significant (*p* < 0.02) during the periods indicated in the figure. JBMD also showed significantly elevated levels of glycerol compared to systemic MD in the first 50 h after ROSC. Further, in the late post-resuscitation period glutamate concentration was significantly higher than the arterial level. In patients with favorable outcome (CPC 1–2). The differences between time-averaged mean JBMD variables and corresponding systemic values were, except for glycerol, statistically non-significant.

The LP ratio in jugular blood remained elevated (> 16) during the first 20 h in both outcome groups as shown in Figure 13 (right panels). After 20 h, an almost complete normalization of the LP ratio was observed. However, the cerebral level of lactate remained high in the CPC 3–5 group (mean level > 2.7 mM), and was paralleled by a marked increase in pyruvate. Based on the biochemical definitions presented previously six patients with unfavorable outcome exhibited ongoing secondary ischemia during altogether 45 h (20%) of the first 24 h of MD monitoring. In the favorable group, three patients displayed a pattern of ischemia during altogether 13 h (13%). Biochemical signs interpreted as mitochondrial dysfunction was noticed in 46% in patients with unfavorable outcome (*n* = 13) and 38% of the time in patients with favorable outcome (*n* = 5).

As discussed previously, a sudden interruption of CBF is instantaneously reflected in a shift in cytoplasmic redox state and a marked increase in cerebral LP ratio (Figure 5B) (44–46, 79). Within a few min, cerebral energy reserve is completely depleted (44–46). If CBF is adequately restored, reperfusion after 30 min of global cerebral ischemia, results in an almost complete normalization of cerebral LP ratio within 90 min (66, 68, 79). However, the level of lactate will remain high and is paralleled by a marked increase in pyruvate (Figures 5A,B) (66, 68, 79). Transient cerebral ischemia is known to cause mitochondrial dysfunction and persistently altered mitochondrial function has been documented after apparently successful resuscitation in experimental cardiac arrest (108, 109).

In the present clinical study, it was for practical reasons not possible to start JBMD and obtain biochemical data until approximately 4–7 h after ROSC. In spite of this delay remaining cerebral ischemia was diagnosed in altogether nine patients during the initial 24 h of MD. The observation contrasts to experiences in experimental studies presented above. In these studies, biochemical signs of remaining ischemia were not observed 90 min after recirculation (67, 79). The finding reveals that under clinical conditions prolonged periods of impaired cerebral perfusion are frequent after ROSC.

Cerebral reperfusion after OHCA is complex and there is a lack of data regarding the 1 h after ROSC (110). The pattern of ischemia and compromised energy metabolism described in the study would probably have been even more pronounced if initiation of JBMD had been possible immediately after ROSC. The study indicates that JBMD should be started within the 1 h to reveal insufficient cerebral recirculation. Efforts to improve outcome after OHCA could then focus on improving cerebral perfusion and energy metabolism during this critical period.

The data obtained in this first explorative, feasibility study have recently been extended in a large randomized study and the intentions of the study have been published (110). In this single-center, randomized, double-blinded trial sixty unconscious patients with sustained return of spontaneous circulation after OHCA were randomly assigned to low (63 mm Hg) or high (77 mm Hg) mean arterial blood pressure target. The primary end-point were the difference in mean LP ratio obtained within 48 h between blood pressure groupsand the secondary end-points were the association between LP ratio and all-cause intensive care unit mortality and the association between LP ratio and survival to hospital discharge with poor neurological function. The results of the study have been analyzed and the study is being prepared for publication (Mölström S et al., in manuscript 2022).

## Concluding remarks

The value of routine cerebral microdialysis is dependent on valid clinical interpretation of the biochemical data displayed bedside. When the information from microdialysis is added to other chemical and physiological data as well as indexes calculated during neurocritical care, very large amounts of data are collected from each patient. It may then seem tempting to process this mass of data with sophisticated statistical methods to reveal correlations between variables with the intention of facilitating biochemical interpretation and support therapeutic choices. As shown in a very large, recent study the information obtained from this approach give little useful information for bedside interpretation (111). The authors emphasized that their modeling was not intended to motivate adoption of LP ratio (or any other microdialysis-derived parameter) as a specific or precise prognostic marker in the clinical management of individual patients.

In this review, we have presented our experiences regarding interpretation of the patterns of variables related to cerebral energy metabolism during routine cerebral microdialysis. Bedside biochemical interpretations should be based on experiences obtained from systematic studies. Interpretations based on occasional clinical observations in various pathological conditions should be questioned if they are not supported by known biochemical patterns documented in systematic experimental and clinical studies.

The recommendations presented above may be summarized in 5 items:

1. Bedside interpretation of biochemical data should be based on principles of cerebral energy metabolism established in animal experiments or controlled, systematic clinical studies. Most of the basic biochemical information has been obtained from analyses of brain homogenates and the difference between these data and later data obtained from microdialysis of cerebral interstitial fluid should be noted.
2. The principles and limitations of the microdialysis technique should be taken into account as well as the analytical precision of the techniques used for routine analyzes.
3. As cerebral energy state depends on aerobic degradation of glucose, bedside analysis and display of the interstitial levels of glucose, pyruvate and lactate give a valid evaluation of cerebral energy metabolism. The calculated, interstitial LP ratio reflects cytoplasmatic redox state. Under clinical conditions, an increase in LP ratio is primarily observed in ischemia, hypoxic hypoxia and mitochondrial dysfunction. The levels obtained for the biochemical variables should be compared to the levels (Mean ± SD) published for normal human brain when utilizing identical microdialysis and analytical techniques. For clinical interpretation it is useful to regard a high LP ratio at a simultaneously very low pyruvate level as cerebral ischemia, and a high LP ratio at a normal or high pyruvate level as indication of cerebral mitochondrial dysfunction.
4. Intracerebral microdialysis reflects biochemical variables in a very narrow zone of the surrounding interstitial fluid. In neurocritical care cerebral lesions are often initially local or regional and the positioning of the microdialysis catheter is therefore crucial. If the catheter is placed in a penumbra zone surrounding a focal lesion an early warning of impending deterioration may direct therapy before signs of deterioration is observed by other techniques. The positioning of the catheter should always be confirmed by CT-scanning. Insertion of more than one intracerebral microdialysis catheter will increase the clinically useful information.
5. Microdialysis of cerebral venous blood in the jugular bulb (JBMD) has shown promise to give information of cerebral energy metabolism under certain conditions. The technique appears to be of clinical value in patients with pronounced global perturbation of cerebral energy metabolism especially when it is difficult or impossible to insert intracerebral microdialysis catheters. In patients resuscitated after cardiac arrest the technique have in recent studies indicated that a poor clinical outcome is related to insufficient cerebral circulation several hours after return of spontaneous circulation.

Finally, for routine clinical use the conventional technique of collecting the microdialysate into microvials at regular time intervals for transport to a bedside analyzer is labor intensive and time consuming. The clinical use of microdialysis would probably increase if the dialysate was analyzed on-line by biosensors and the results immediately displayed on a bedside monitor.

## Author contributions

The microdialysis technique was invented and developed by UU. The biochemical background was mainly based on previous experimental studies by C-HN. The clinical studies were mainly conducted at Odense University Hospital and were mainly designed, performed and interpreted by TN, AF, SM, PT, and C-HN. The experimental studies on microdialysis of the draining cerebral blood flow were mainly designed, performed and interpreted by RJ, TN, and C-HN at Odense University Hospital, Denmark. All authors have contributed equally to the design, composition, and writing of this comprehensive review.

## Conflict of interest

The authors declare that the research was conducted in the absence of any commercial or financial relationships that could be construed as a potential conflict of interest.

## Publisher's note

All claims expressed in this article are solely those of the authors and do not necessarily represent those of their affiliated organizations, or those of the publisher, the editors and the reviewers. Any product that may be evaluated in this article, or claim that may be made by its manufacturer, is not guaranteed or endorsed by the publisher.
